# Celastrol increases osteosarcoma cell lysis by γδ T cells through up-regulation of death receptors

**DOI:** 10.18632/oncotarget.12756

**Published:** 2016-10-19

**Authors:** Zhaoxu Li, Junzhe Zhang, Jicun Tang, Ruiying Wang

**Affiliations:** ^1^ Department of Orthopaedics, Affiliated Hospital, Guilin Medical University, Guilin, Guangxi, China

**Keywords:** osteosarcoma, tripterine, TNF-related apoptosis-inducing ligand, immunotherapy

## Abstract

γδ T cells has been shown to exhibit profound antitumor effects in a broad range of tumor entities, including OS. However, resistance to γδ T cells is a serious problem in the management of OS. This study investigates the impact of celastrol on the expression of death receptors 4/5 (DR4/5) on OS cell lines (HOS, U2OS) and cancer cell lysis by γδ T cells. The results showed that celastrol increased transcription of DR4/5 in HOS and U2OS, leading to increased cell surface, and total DR4/5 protein expression. Celastrol sensitizes OS cell lines or autologous OS cells to healthy donors-derived or OS patient-derived γδ T cell cytotoxicity *in vitro*. The induction of DR4/5 molecules increased lysis of HOS and U2OS by γδ T cells which was abolished by addition of a blocking TRAIL antibody. Importantly, the cytotoxic activity of γδ T cells was unaltered by small-dose celastrol. Taken together, our data show that celastrol up-regulated DR4/5 on OS cells to be responsible for intercellular TRAIL/APO-2L crosslink that confers increased cancer cell lysis by γδ T cells. These results suggest the clinical evaluation of celastrol in OS, especially in combination with immunotherapy approaches employing adoptive γδ T cell transfer.

## INTRODUCTION

Osteosarcoma (OS) is the most common malignant bone tumor in children and adolescents [1]. Although survival improved initially in a drastic fashion with systemic chemotherapy and local control surgery, recent decades have seen little to no further gains in this area [2]. At present, the use of additional immunotherapy such as adoptive cell therapy (ACT) may have definitively improved the survival of patients with OS [3, 4]. Nonetheless, ACT is severely limited by the lack of suitable targets exclusive to cancer, although it has been successful in the treatment of hematologic malignancies and some solid cancers [5].

γδ T cells, exhibiting potent MHC unrestricted lytic activity, contribute to the elimination of virus-infected cells as well as to antitumor immune responses [6]. Our previous studies revealed that γδ T cells were capable of mediating cytotoxicity against OS cells [7–9], suggesting their potential utility as anti-OS therapy, although γδ T cell-based immunotherapeutic strategies in this area are as yet of limited success [10]. However, accumulating evidence suggests that γδ T cell immunotherapy needs to be combined with additional strategies such as chemotherapeutics for improved clinical results [11].

TRAIL has been hailed as a promising new therapy for cancers, because that it induces apoptosis in cancer cells while sparing most normal cells [12]. Among five different receptors, only death receptors 4/5 (DR4/5) contain a functional cytoplasmatic death domain motif and are capable of delivering the apoptotic signal of TRAIL/Apo-2L [13]. Previous reports have shown that most human cancer cell types that have been tested are sensitive to the apoptotic effects of TRAIL both *in vitro* and *in vivo* [14, 15]. However, many OS cells respond poorly to the cytotoxic effects of TRAIL alone [16]. Thus agents that can modulate the mechanism of resistance to TRAIL have a potential in improving the treatment of OS.

A series of experiments have demonstrated celastrol, isolated from traditional Chinese medicine Tripterygium wilfordii, possesses a wide spectrum of potent antitumor activity [12, 17–21]. Recently, two studies have shown that celastrol induced apoptosis and autophagy via the ROS/JNK signaling pathway and the mitochondrial apoptotic pathway in human OS cells [22, 23]. In addition to these capabilities, celastrol has also been shown to sensitize cancer cells to TRAIL-induced apoptosis by up-regulation of DR4/5 [12, 17, 19]. However, the functional consequences of celastrol treatment for cellular immunity remain unclear.

Here, we wanted to assess whether celastrol was capable of up-regulating the expression of DR4/5 on OS cells and increasing lysis of OS cell by γδ T cells. Our data showed that celastrol increased transcription of DR4/5 in OS cell lines (HOS, U2OS), leading to increase of cell surface, and total DR4/5 protein expression. And, celastrol sensitizes OS cell lines or autologous OS cells to healthy donors-derived or OS patient-derived γδ T cell cytotoxicity *in vitro*. Furthermore, the induction of DR4/5 molecules increased lysis of OS cell lines (HOS, U2OS) by γδ T cells from healthy donors, which was abolished by addition of a blocking TRAIL antibody. Importantly, the cytotoxic activity of γδ T cells was unaltered by small-dose celastrol. Overall, these data indicated that celastrol induced upregulation of DR4/5 on OS cells to be responsible for intercellular TRAIL/APO-2L crosslink that confers increased cancer cell lysis by γδ T cells.

## RESULTS

### Celastrol induces the mRNA of the DR4 and DR5 in the OS cell lines

**Figure 1:**
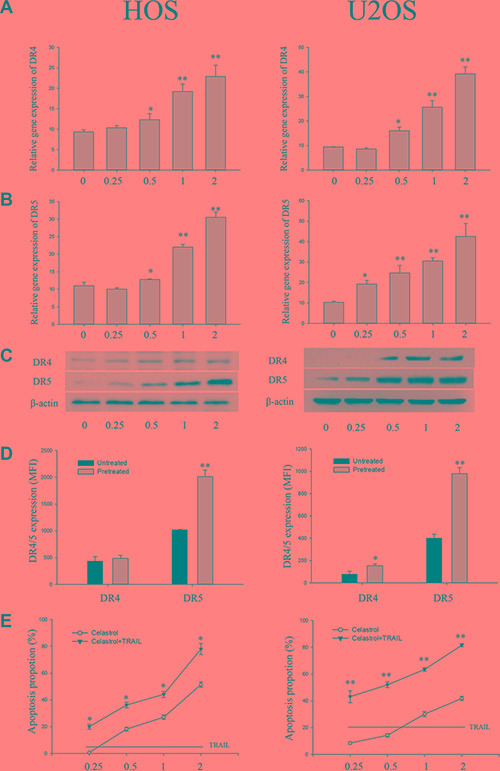
Impact of celastrol on DR4 and DR5 expression in OS cell lines (HOS and U2OS) (**A**, **B**) celastrol induces DR4 and DR5 gene mRNA expression. OS cell lines (HOS and U2OS) cells (10^6^ cells/well) were treated with various concentrations of celastrol for 24 h, and total RNA was extracted and examined for expression of DR4 and DR5 by RT-PCR. (**C**) OS cell lines (HOS and U2OS) cells (5 × 10^5^ cells/well) were treated with various concentrations of celastrol for 24 h. Whole cell extracts were then prepared and analyzed for DR4 and DR5 expression by Western blotting. β-actin was used as an internal control. (**D**) Impact of celastrol on cell surface expression of DR4 and DR5. Cell surface expression of DR4 and DR5 was measured by flow cytometry on OS cell lines (HOS and U2OS) cells following 1 μmol/L celastrol treatment for 24 h. DR4 and DR5 expression in MFI was gated on osteosarcoma cell lines, HOS and U2OS, respectively. (**E**) Celastrol sensitizes OS cell lines (HOS and U2OS) to TRAIL. Cells (5000 cells/well) were pretreated with various concentrations of celastrol for 24 h, washed with PBS to remove celastrol, and then cells were treated with or without TRAIL (100 ng/ml) for 24 h. The cell viability was determined by flow cytometry. Results are presented as mean ± SD, *n* = 3. *P < 0.05, **P < 0.01 vs. corresponding control group; one-way ANOVA plus post hoc Dunnett test.

To determine whether TRAIL receptors are induced by celastrol at the transcriptional level, the mRNA of the DR4 and DR5 in the OS cell lines HOS and U2OS was firstly investigated by RT- PCR. The results revealed that the mRNA of the DR4 and DR5 were constitutively present at low levels in the OS cell lines HOS and U2OS. Treatment with celastrol for 24 h increased both DR4 and DR5 mRNA levels in a dose-dependent manner, respectively, whereas the housekeeping gene β-actin did not change significantly (Figure 1A–1B).

### Celastrol increases the DR4 and DR5 protein levels in the OS cell lines

To determine whether celastrol-induced changes in TRAIL receptors mRNA were mirrored by altered protein expression, we then did western blot analysis with the human OS cell lines HOS and U2OS. Using different antibodies that recognizes both DR4 and DR5, Very weak or no binding was detected on human OS cell lines HOS and U2OS (Figure 1C, left). Treatment with celastrol for 24 h, DR4 and DR5 protein levels were also enhanced up-regulation in both HOS cells and U2OS cells in a dose -dependent manner (Figure 1C, right). U2OS cells exposed to celastrol (1 μM) for 24 h, the DR5 expression increased about 5.6-fold; whereas in HOS cells, celastrol (1 μM) treatment for 24 h caused about 3.3-fold increment. Similarly, DR4 protein levels were also enhanced by celastrol in HOS and U2OS cells (Figure 1C, right).

### Celastrol treatment increases surface expression of the DR4 and DR5 on the OS cell lines

To investigate whether changes of TRAIL receptors expression could be observed on the OS cell lines cell surface where TRAIL signaling takes place, the surface expression of the DR4 and DR5 were investigated by flow cytometry. TRAIL receptors expression was quantified by calculation of mean fluorescence intensity (MFI). Overall, constitutive expression of DR5 was higher on HOS and U2OS cells compared with DR4 (Figure 1D). Both DR4 and DR5 were constitutively expressed on HOS and U2OS cells. After 24 h of incubation with celastrol (1 μM), in U2OS cells, a 2.5-fold increase of DR5 and a 1.6-fold increase of DR4 surface expression were observed, whereas HOS cells revealed a 2.1-fold increase in DR5 with no obvious changes (1.1-fold) in DR4 surface expression (Figure 1D).

### Celastrol -induced DR4 and DR5 up-regulation is biologically active

We then asked whether up-regulation of DR4 and DR5 expression by celastrol is functionally relevant and determines the responsiveness of OS cells to TRAIL-induced apoptosis. To address this point, we pre-treated OS cells with celastrol for 24 h to up regulate DR4 and DR5 levels and then added TRAIL to trigger apoptosis for a further 24 h. As shown in Figure 1E, pre-treatment with celastrol significantly enhanced TRAIL-mediated apoptosis in human OS cell lines HOS and U2OS in a dose -dependent manner. By comparison, no sensitization for TRAIL-induced apoptosis was found when OS cell lines HOS and U2OS were pre-treated without celastrol, showing that pre-treatment with celastrol to up regulate DR4 and DR5 expression was required to render OS cells susceptible to TRAIL.

### OS cells pre-treated with celastrol induce activation of γδ T cells under co-culture conditions

Peripheral blood mononuclear cells (PBMC) from healthy donors (*n* = 4) were stimulated once with zoledronate (Zol) and cultured in presence of IL-2. After 2 weeks of culture, γδ T cells from healthy volunteer were selectively expanded, similar to our previous reported data [7].

**Figure 2:**
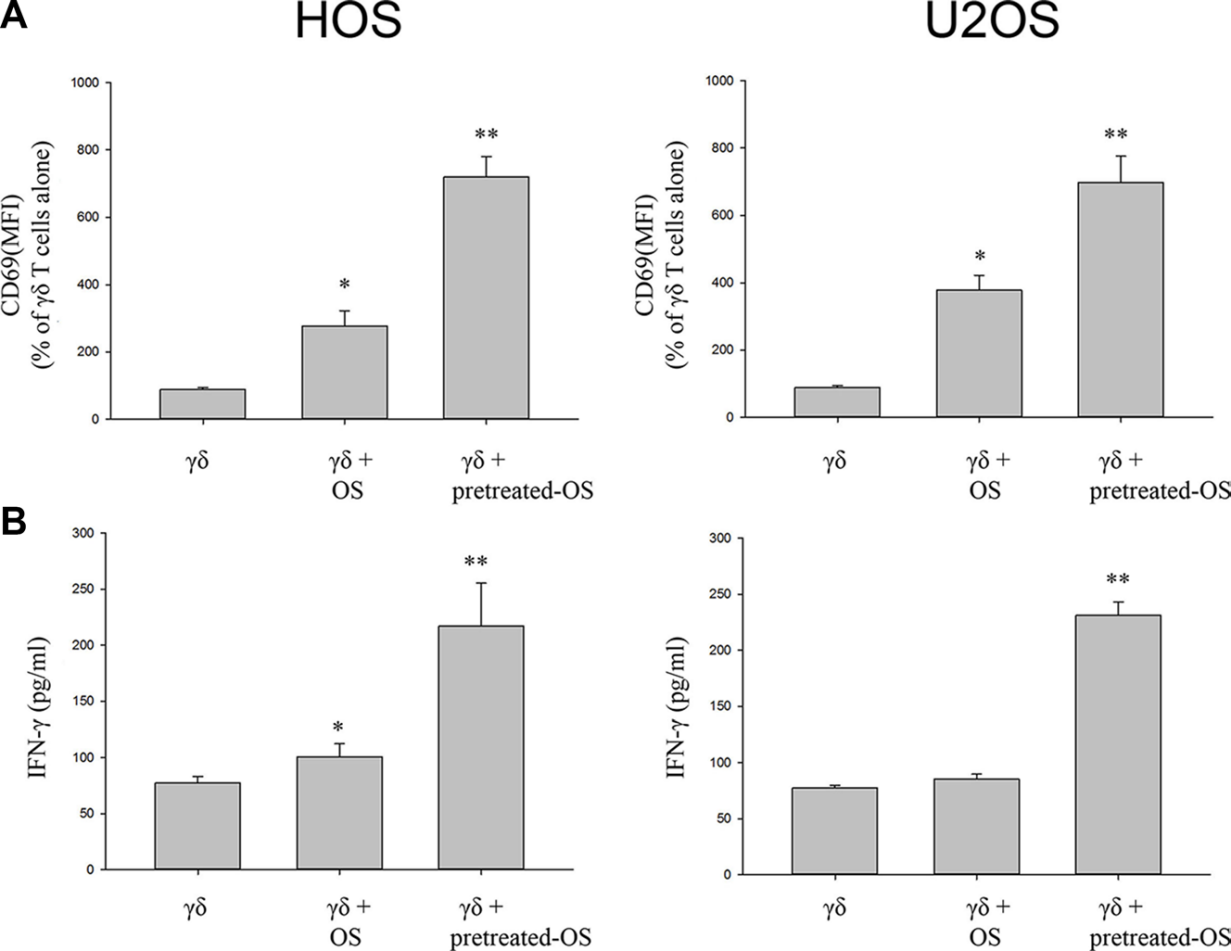
Celastrol-treated OS cells increase CD69 expression and the proportion of IFNγ-producing by γδ Tcells from healthy donors (**A**) Celastrol-treated OS cells increase CD69 expression by γδ T cells from healthy donors. (**B**) Celastrol-treated OS cells increase IFNγ production by γδ T cells from healthy donors. Quantification of CD69 expression on γδ T cells and IFNγ production by γδ T cells alone or γδ T cells following a 1 day culture with OS cell lines HOS and U2OS, or OS cells pre-treated with 1 μM celastrol for 24 h, prior to addition of γδ T cells, at a T-cell: OS cell ratio of 3:1. Following the incubation period, CD69 expression and IFN-γ release were measured as detailed in Section 4. Representative histograms showing CD69 expression on γδ T cells and IFNγ production by γδ T cells alone, or in the presence of OS cells, or OS cells pre-treated with celastrol. Data shown are the mean+SEM of four independent experiments, performed in duplicate, from different donors. (*n* = 4, *P < 0.05, **P < 0.01 compared to T cells alone).

In order to determine whether OS cells could induce activation of γδ T cells, we determined the early activation marker CD69 on γδ T cells using flow cytometry analysis. When γδ T cells were co-cultured with OS cell lines HOS and U2OS for 24 h a significant increase in CD69 expression was observed in the γδ T cells (*P* < 0.05) (Figure 2A). Following co-culture with OS cell lines HOS and U2OS pre-treated with celastrol for 24 h, CD69 expression was further increased on γδ T cells, and this was statistically different from untreated OS cell lines (*P* < 0.01) (Figure 2A).

### OS cells pre-treated with celastrol induce IFNγ production by γδ T cells

We have previously shown that IFN-γ pretreated OS targets stimulated γδ T cells to secrete IFN-γ [7].We therefore determined if co-culture with OS cells pre-treated with celastrol induce the production of IFNγ by γδ T cells. Co-culture with OS cells HOS or U2OS pre-treated with celastrol significantly increased IFNγ production of γδ T cells (*P* < 0.01), from (100.9 ± 11.4) pg/ml to (216.9 ± 38.4) pg/ml, or (85.2 ± 4.6) pg/ml to (231.2 ± 12.0) pg/ml, respectively (Figure 2B). Interestingly, there was no enhanced production of IFNγ following co-culture of γδ T cells with untreated U2OS (*P >* 0.05).

### Celastrol sensitizes OS cell lines to healthy donors’ γδ T cell cytotoxicity *in vitro*

**Figure 3:**
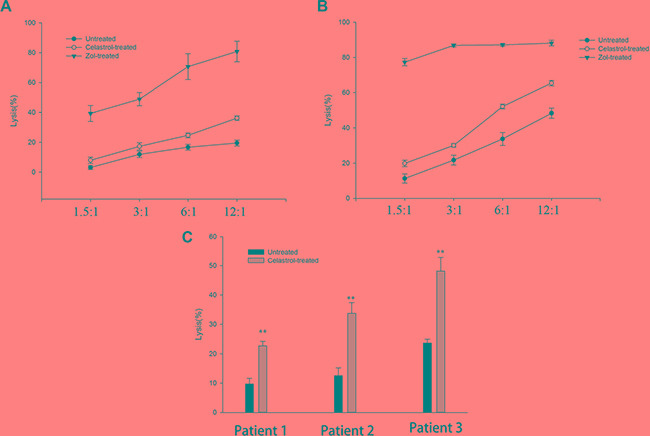
Celastrol sensitizes OS cells to γδ T cells lyses (**A**) Celastrol sensitizes HOS to γδ T cells from healthy donors lyses. (**B**) Celastrol sensitizes U2OS to γδ T cells from healthy donors lyses. (**C**) Cytotoxic activity of OS patient-derived γδ T cells against autologous OS cancer cells. γδ T cells were expanded from a patient with newly diagnosed OS for 2 W, using Zol and IL-2. In parallel, autologous OS cells were propagated in monolayer culture in a six-well plate. Two different target OS cell lines HOS and U2OS treated with or without Celastrol (1 μM) or for Zol (1 μM) 24 h were tested for their sensitivity to γδ T cell obtained and used at various E:T ratios, and autologous OS cells treated with or without Celastrol (1 μM) 24 h were tested for their sensitivity to OS patient-derived γδ T cell obtained and used at 1:6 of E:T ratios. Results indicate cytotoxicity of tumor targets following 4 hrs co-culture with γδ T cells. Data are mean percentage ± SEM of 4 different experiments, each carried out in triplicate. *P < 0.05, **P < 0.01 vs. corresponding control group; Student's *t* test.

To address the impact of celastrol on γδ T cell-mediated OS cell lysis, OS cell lines, HOS and U2OS, that were incubated with 1 μM celastrol for 24 h were subsequently co-cultured with γδ T cells at a defined E:T ratio (see Materials and Methods). Following a 6-h incubation, tumor cell lysis was measured by detection of lactate dehydrogenase (LDH) in the supernatant. As shown in Figure 3A–3B, similar to our previous reported data [7, 8], γδ T cells induced by Zol exhibited from little to moderate cytotoxic activity OS cell lines, HOS and U2OS. Following co-culture with OS cell lines, HOS and U2OS, pre-treated with celastrol for 24 h, γδ T cell cytotoxicity was further increased, and this was statistically different from untreated OS cell lines (*P* < 0.01) (Figure 3A, 3B).

### Celastrol sensitizes autologous OS cells to OS patient-derived γδ T cell cytotoxicity *in vitro*

Next, we evaluated the cytotoxic activity of patient-derived γδ T cells against autologous OS tumor cells. γδ T cells from OS patients (*n* = 3) were selectively expanded, similar to our previous reported data [8]. The results showed that untreated autologous OS cells were poorly sensitive to γδ T cell cytotoxicity. Lyses percentages ranged from (9.7 ± 1.9) % to (23.7 ± 1.3) %, at an E: T ratio of 6:1. But, all these cells showed significantly higher cytotoxicity against autologous OS cells after treatment with Celastrol (Figure 3C).

### Up-regulation of DR4 and DR5 expression following celastrol treatment enhances γδ T cell lysis of OS cells

**Figure 4:**
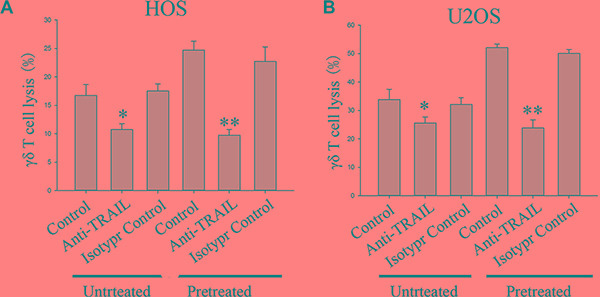
Effect of a neutralizing TRAIL antibody on cytotoxic lysis of OS cells by γδ T cells from healthy donors HOS (**A**) and U2OS (**B**) were incubated with vehicle or 1 μM celastrol for 24 h. Before starting cytotoxicity assay cancer cells were pre-incubated with an TRAIL antibody (1 μg/ml) for 1 h. An isotype control antibody (1 μg/ml) was used as negative control. Values are means ± SEM of *n* = 4 experiments. *P < 0.05, **P < 0.01; one-way ANOVA plus post hoc Bonferroni test.

DR4 and DR5 stimulation by specific ligands such as TRAIL has been shown to enhance innate antitumor immunity including NK cell–mediated lysis of tumor cells [24]. Therefore, to confirm a causal link between celastrol -induced up regulation of DR4 and DR5 protein expression and the concomitant increase of γδ T cell-mediated tumor cell lysis by celastrol, a neutralizing antibody to TRAIL was tested for its inhibitory action on OS cell lysis. The results showed that γδ T cells lysed untreated HOS and U2OS cells up to 17% and 34% at an E:T ratio of 6:1, respectively (Figure 4). Celastrol (1 μM) treatment for 24 h markedly enhanced the OS cell susceptibility to γδ T cell mediated killing, resulting in a statistically significant increase of HOS and U2OS lysis (Figure 4). This increase in cytotoxicity was critically dependent on Apo2L/TRAIL interaction, because it was completely abolished by addition of a blocking TRAIL mAb, whereas addition of an isotype control mAb did not significantly decrease γδ T -mediated lysis of celastrol -treated human OS cell lines HOS and U2OS cells (Figure 4).

### Small-dose celastrol does not interfere with γδ T cell function

**Figure 5:**
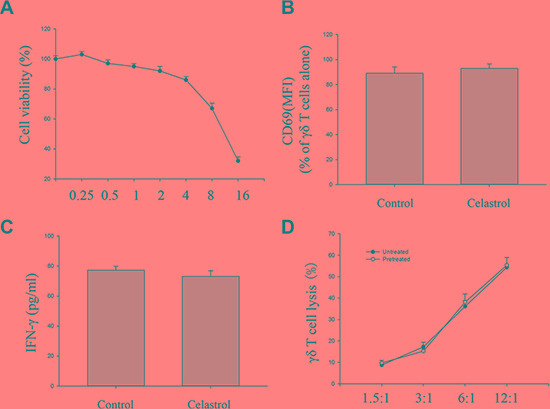
Impact of celastrol on cytotoxic activity of γδ T cells (**A**) γδ T cells (5000 cells/well) were incubated various concentrations of celastrol. After 24 h, cell viability was determined by the MTT assay. Viability of control cells was set at 100%, and viability relative to the control is presented. (**B**) Impact of celastrol on cell surface expression of CD69 expression by γδ T cells. Cell surface expression of CD69 was measured by flow cytometry on γδ T cells following with or without 1 μM celastrol treatment for 24 h. CD69 expression in mean fluorescence intensity (MFI) was gated onγδ T cells. (**C**) Impact of celastrol on IFNγ production by γδ T cells. Quantification of CD69 expression on γδ T cells and IFNγ production by γδ T cells alone or γδ T cells following 1 μM celastrol treatment for 24 h. (**D**) Impact of celastrol on the cytotoxic effect of γδ T cells. Daudi cells following 4 h co-culture with γδ T cells pre-treated with 1 μM celastrol for 24 h at various E:T ratios, cytotoxicity was determined by the LDH assay. Values are means ± SEM, *n* = 4 (4 donors). Student's *t* test.

To determine whether celastrol does interfere with γδ T cell function, viability tests were firstly performed by the MTT assay after incubating γδ T cells with various concentrations of celastrol for 24 h. As compared to control group (100% viability), incubation of γδ T cells with less than 4 μM celastrol yielded at least viability rates of 86% ± 2.3% (all values as means ± SEM of *n* = 4 experiments) (Figure 5A). These findings implied the function of γδ T cells to probably be unaltered by small-dose celastrol. We therefore determined whether co-culture with small-dose celastrol (1 μM) for 24 h could not interfere with the activation and the cytotoxic activity of γδ T cells. The results showed that whereas treatment with 1 μM celastrol for 24 h did not change CD69 expression and IFNγ production of γδ T cells, and the γδ T cell sensitive Daudi cell susceptibility to γδ T cell mediated killing (Figure 5B–5D). Taken together, the results obtained from these experiments revealed no influence of a pretreatment of γδ T cells with small-dose celastrol (1 μM) on γδ T cell-mediated tumor cell lysis, implying the cytotoxic activity of γδ T cells to be unaltered by small-dose celastrol (1 μM).

## DISCUSSION

In the present study, we show that celastrol increased DR4 and DR5 expression on OS cell lines HOS and U2OS, and sensitized OS cells to TRAIL-induced apoptosis by up-regulation of DR4/5. More importantly, celastrol increased of γδ T cell-mediated lysis of OS cells. At last, the cytotoxic activity of γδ T cells to be unaltered by small-dose celastrol. Thus, our findings suggest that celastrol enhance γδ T cell mediated lysis of OS cells by upregulation of DR4 and DR5 on the surface of OS cells.

γδ T cells are cytotoxic through a range of mechanisms that lead to antitumor activity, including via induction of apoptotic cell death by the TRAIL pathway [8]. Yet, resistance to TRAIL/APO-2L-mediated apoptosis is a great challenge for the treatment of OS by TRAIL/APO-2L [25]. Our results show that both HOS and U2OS are resistant to the proapoptotic effect of TRAIL, in agreement with previous studies [26]. Thus, agents that can modulate the mechanism of resistance to TRAIL have a potential in improving the effect of ACT for OS.

Celastrol and TRAIL/APO-2L exerted synergistic anti-proliferative effect against human cancer cells [17, 19]. This effect was critically correlated to DR4 and DR5 expression levels at the cancer cell surface [12]. In our study, celastrol increased transcription of DR4/5 in HOS and U2OS, leading to increased cell surface, and total DR4/5 protein expression. More important, celastrol -induced DR4 and DR5 up-regulation is biologically active because that pre-treatment with celastrol significantly enhanced TRAIL-mediated apoptosis in human OS cell lines HOS and U2OS in a dose -dependent manner.

In order to determine whether OS cells pretreated with celastrol could induce activation of γδ T cells, it was assessed by IFNγ production and CD69 expression. Our study reveals that co-culture with OS cells or OS cells pretreated with celastrol induces an enhanced IFNγ production and CD69 expression in γδ T cells *in vitro*. While the formal demonstration of interactions between γδ T cells and OS cells has yet to be demonstrated, our study highlights a potentially intriguing capacity of OS cells pretreated with celastrol to influence γδ T cell function. The results of this study showed that OS cells pretreated with celastrol are capable of inducing greater numbers of functionally active γδ T cells, implying potentially γδ T cell-mediated cytotoxic effects on OS cells themselves *in vitro*.

We next determined the significance of enhanced DR4 and DR5 surface expression following celastrol treatment for OS cells for γδ T cell cytotoxicity using γδ T cells from PBMCs of healthy volunteer stimulated with Zol. In our study, γδ T cells exhibited from little to moderate cytotoxic activity OS cell lines, HOS and U2OS, and consistent with our previous studies [7, 8]. However, this lysis was significantly enhanced following treatment of cells with celastrol. The cytotoxocity was critically dependent on Apo2L/TRAIL interaction, because it was abolished by addition of anti- TRAIL. This shows that DR4 and DR5 expression renders OS cells susceptible for γδ T cell–mediated killing that can be markedly enhanced by celastrol treatment.

Patients with newly diagnosed cancer had somewhat lower circulating numbers of γδ T cells than did healthy donors [27]. Nonetheless, these cells from OS patients could be expanded efficiently and were functionally competent [8], although autologous OS cells were poorly sensitive to γδ T cell cytotoxicity. But, all γδ T cells populations showed significantly higher cytotoxicity against autologous OS cells after treatment with Celastrol. Thus, ACT using patient-derived γδ T cells combined with additional strategies represents one natural extension of the preclinical approach of OS described in this article. To expand these investigations, of course, further consideration should be taken to xenograft OS model in future study.

Increasing chemotherapy doses overcome tumor resistance to immune cells, but are toxic and often only partly effective [28]. Given the possible toxicologic effect of celastrol, we further addressed whether celastrol exhibited inhibitory activities on γδ T cells. The results of this study showed that whereas treatment with 1 μM celastrol for 24 h did not change CD69 expression and IFNγ production, and the cytotoxic effect of the γδ T cells, suggesting that γδ T cells could not be infused after small-dose celastrol treatment. Previous studies indicated that sequential, but not simultaneous, administration of chemotherapy and immune effector cell led to synergistic cytotoxicity, because that tumor targets were pretreated with chemotherapy agents to allow time for chemotherapy to sensitize tumor cells [7, 28]. Therefore, this sequence of exposure was used in present study to assess whether celastrol was capable of increasing lysis of OS cell by γδ T cells, although γδ T cells could not be infused after small-dose celastrol treatment.

In summary, the present study identified upregulation of DR4 and DR5 expression in OS cells leading to γδ T cell-mediated OS cell lysis as a novel antitumorigenic mechanism of celastrol, indicating that celastrol might be able to prime OS cells *in vivo* for γδ T cell lysis and even open up the possibility of an adoptive immunotherapy of OS after celastrol treatment using γδ T cells.

## MATERIALS AND METHODS

### Materials

Celastrol was obtained from Sigma-Aldrich (Saint Louis, Missouri), Zol from Novartis Pharmaceuticals (Basel, Switzerland), and rhIL-2 from Primegene Bio-Tech Co. (Shanghai, China), respectively. The following FITC- or PE –conjugated mAbs were obtained from eBioscience (San Diego, CA): anti-TCR-γδ, anti-CD3, anti-CD69, anti-DR4 (DJR1), and anti-DR5 (DJR2-4) mAbs were obtained from eBioscience (San Diego, CA). Additionally, the purified anti-human TRAIL-R and TRAIL were from BD Bioscience (San Diego, CA), Mouse monoclonal anti-β-actin antibody was purchased from Sigma-Aldrich (Saint Louis, Missouri). Isotype-matched monoclonal antibodies were obtained from BD biosciences and eBioscience and used as staining controls. 3-(4,5-dimethylthiazolyl-2)-2,5-diphenyltetrazolium bromide (MTT), RNase were purchased from Sigma (St. Louis, USA), Annexin-V/PI binding assay kit was from Invitrogen Ltd. (Paisley, UK), the CytoTox 96 Non-Radioactive Cytotoxicity Assay from Promega (Madison, WI), and IFNγ ELISA kits from Bender MedSystem (Vienna, Austria). Other chemicals were from Beyotime Biotechnology Company (Shanghai, China).

### Cell cultures

Daudi, Raji, human osteosarcoma cell lines, HOS and U2OS, and Human osteoblast cell line, hFOB, were obtained from the Cell Collection of Chinese Academy of Science (Shanghai, China). Primary osteosarcoma cells were derived from patients with OS. HOS, U2OS cells and primary cells were cultured in DMEM medium, Daudi and Raji cells were grown in RPMI 1640 medium, and hFOB cells in DMEM/F12 medium, at 37°C in a humidified atmosphere, containing 5% CO2. All procedures involved clinical specimens were approved by Human Research Ethics Committees of the Affiliated Hospital of Guilin Medical University (Guilin, China).

### Generation of γδ T cells

Our study was approved by Human Research Ethics Committees of the Affiliated Hospital of Guilin Medical University (Guilin, China). After obtaining consents from healthy donors and OS patients, human peripheral blood (10 ml) was collected. PBMC were isolated from fresh blood of four different healthy donors, and γδ T cells were expanded as described our previous study [7, 29]. The purity of the expanded γδ T cells was assessed by flow cytometry. Fold expansion rate of γδ T cells was calculated as follows: fold expansion rate of γδ T cells = absolute number of γδ T cells at the end of the culture)/(absolute number of γδ T T cells at day 0 of culture) [30]. In all functional assays, the purity of γδ T cells was > 92%.

For some experiments fractions of γδ T cells were treated with PBS or celastrol. In this case PBS or celastrol was added to γδ T cell suspension into the culture flask 24 h before starting γδ T cell cytotoxicity assay.

### IFN-γ eLISA

γδ T cells exposed to complete media alone or containing celastrol for 24 h, or incubated with OS cells or OS cells pre-treated with celastrol at an E: T ratios of 3:1. After 6 h co-culture, supernatants were collected, and the production of IFN-γ was determined using commercially available ELISA kits.

### Cytotoxicity assays and blocking experiments

*Ex vivo* expanded γδ T cells were purified and their cytotoxic activity against cancer cell lines was measured by a standard lactate dehydrogenase release assay as described our previous study [8, 9].

Experiments to determine the functional involvement of TRAIL in enhanced γδ T cell-mediated tumor cell killing were performed using a neutralizing antibody against TRAIL. Prior to coculture in blocking experiments, γδ T cells were incubated with functional grade anti-human TRAIL (RIK-2) (eBioscience) at 10 μg/ml or an isotype control antibody as negative control for 2 h, to block TRAIL-mediated cytotoxic pathways [31].

### Quantitative real-time polymerase chain reaction (PCR)

Quantitative real-time PCR was performed with SYBR green detection using the iCycler (Bio-Rad, Hercules, CA, USA) as described previously [7]. Gene specific primer sequences were as follows: for human-DR4, forward CTGAGCAACGCAGACTCGCTGT CCAC, reverse TCCAAGGACACGGCAGAGCCTGT GCCAT; for human-DR5, forward AAGACCCTTGTGC TCGTTGTC, reverse GACACATTCGATGTCACTCCA. β-actin was used as an internal control to standardize mRNA levels. Specificity of the expected products was demonstrated by melting curves analysis. PCR reactions for each sample were done in triplicate. The real-time PCR data were quantified using the ΔCT method with the formula: *n* = 100 * 2^−(^^ΔCT targeted gene−ΔCT β-Actin)^.

### Western blot analysis

OS cell lines (HOS and U2OS) cells (5 × 10^5^ cells/well) were treated with complete media alone or containing various concentrations of celastrol for 24 h. To determine the DR4 and DR5 protein levels in the OS cell lines, we prepared whole cell extracts as described previously [22].

### Flow cytometry

Flow cytometric analysis was performed as our previously described [8]. In brief, target cells were suspended in PBS, and incubated with antibody or isotype-matched antibody for 30 min at 4°C. After washing with PBS, Samples were analyzed by flow cytometry (FACScan, Becton Dickinson) equipped with a CellQuest software (BD biosciences; San Jose, CA).

### Annexin-V/PI binding assay

Cells (10000 cells/well) were pretreated with various concentrations of celastrol for 24 h, washed with PBS to remove celastrol, and then cells were treated with or without TRAIL (100 ng/ml) for 24 h. Then, cells harvested by trypsinization were used for Annexin-V/PI binding assay as our described previously [7]. At last, cells were harvested and washed with PBS, and incubated with 5 μl of FITC-Annexin V and 1 μl of PI working solution for 30 min in the dark at 4°C. After washed, apoptotic cells were immediately analyzed by flow cytometry.

### Cell proliferation assay

The cell proliferation was determined by the MTT assay. γδ T cells were seeded into 96-well plates and cultured overnight; exposed to complete media alone or containing various concentrations of celastrol for 24 h. 20 μl of MTT solution (5 mg/ml) was then added into each well and incubated for 4 hours. After a 4-h incubation, 150 μl of DMSO were added per well to dissolve the formazan crystal. An enzyme-linked immunosorbent assay reader (Molecular Device Inc, Silicon Valley, CA) was used to measure the absorbance at 490 nm. Each experiment was repeated in triplicate. The viability rate of γδ T cells was calculated for each well as: A490 treated cells/A490 control cells × 100%.

### Statistics

Values were expressed as mean ± SEM, and statistical analysis was performed by using Students *t* test or with one-way ANOVA plus post hoc Bonferroni or Dunnett test, and the results were considered significant when values of *P* < 0.05.
